# Placebo and nocebo effects and operant pain-related avoidance learning

**DOI:** 10.1097/PR9.0000000000000748

**Published:** 2019-06-07

**Authors:** Thomas Janssens, Ann Meulders, Bien Cuyvers, Luana Colloca, Johan W.S. Vlaeyen

**Affiliations:** a Health Psychology, KU Leuven (University of Leuven), Leuven, Belgium; b Research Group Behavioral Medicine, Maastricht University, Maastricht, the Netherlands; c Department of Pain Translational Symptom Science, School of Nursing, University of Maryland, BaltimoreMD, USA; d Department of Anesthesiology/Psychiatry, School of Medicine, University of Maryland, Baltimore, MD, USA; e Center to Advance Chronic Pain Research, University of Maryland, Baltimore, MD, USA

**Keywords:** Avoidance, Operant learning, Placebo, Nocebo, Expectations

## Abstract

**Introduction:**

Research on learning in placebo and nocebo has relied predominantly on Pavlovian conditioning procedures. Operant learning procedures may more accurately model learning in real-life situations in which placebo and nocebo effects occur.

**Objectives:**

To investigate the development and persistence of placebo and nocebo effects using an operant avoidance learning task.

**Methods:**

Pain-free participants (n = 58) could learn to avoid pain by performing movements that differed in difficulty and intensity of painful stimulation. Participants performed movements in 2 contexts. In the high cost of avoidance context, pain stimulus intensity reduced with increasing movement difficulty. In the low cost of avoidance context, contingencies were reversed. Participants rated pain expectations and pain intensity. During test, movement difficulties were unchanged, but participants always received a medium-intensity pain stimulus. Placebo and nocebo effects were defined as lower/higher pain intensity ratings for trajectories that previously resulted in low/high-intensity compared with medium-intensity stimulation.

**Results:**

As expected, participants acquired differential movement-pain expectations and differential movement choices. Testing with a medium-intensity pain stimulus quickly erased differences in movement choice across contexts, but differences in pain expectations were maintained. Pain modulation across context was in line with movement-pain expectations. However, we only observed placebo effects within the low cost of avoidance context and found no evidence of nocebo effects.

**Conclusion:**

Operant learning can change pain expectations, pain modulation, and pain-related avoidance behavior. Persisting pain expectations suggest that acquired pain beliefs may be resistant to disconfirmation, despite self-initiated experience with novel pain-movement contingencies.

## Abstract 2

Supplemental Digital Content is Available in the Text.

## 1. Introduction

Placebo effects occur when treatment-related cues are presented with an inert medical treatment, resulting in an improved clinical state. Conversely, nocebo effects occur when these cues result in a worsened clinical state.^5^ Research on placebo and nocebo mechanisms in pain has convincingly shown that positive or negative expectations can modulate self-reported pain intensity.^6,29,34^ Furthermore, classical or Pavlovian conditioning—in which visual or somatosensory cues are paired with an outcome to elicit a conditioned response when presenting the cue in absence of the outcome—has been used to elicit pain modulation, both in combination with direct instruction of treatment expectations,^7,34^ as well as without any co-occurring information,^21^ suggesting a key role for learning in pain modulation.

Learning also is essential in the development and exacerbation of pain-related behavior. Previous research has demonstrated that after pairing a neutral cue with a painful stimulus, this initially neutral stimulus may start to elicit conditioned fear responses.^24,25^ Furthermore, operant procedures—in which voluntary behavior is shaped based on its consequences—have been used to study avoidance behaviors, their role in the maintenance of fear of movement-related pain,^23^ and their association with pain intensity and chronic pain.^35^

However, little is known as to whether operant pain-related behaviors also modulate pain.^16,36^ In the current study, we therefore adapted an existing operant learning task^23^ to investigate the effect of operant pain-related behavior on placebo and nocebo effects. In this task, participants freely perform different reaching movements and can avoid pain by performing more effortful movements. Our adaptation consisted of manipulating the intensity of the pain stimulus (using a high-, medium-, or low-intensity stimulus) contingent upon movement effort. In a within-subject design, in 1 context (similar to the original task), participants could avoid the high-intensity pain stimulus by choosing movement trajectories that needed more effort, creating a high cost of avoidance. In the other context, movement trajectories remained the same, but the trajectory-pain contingencies were reversed, creating low cost of avoidance. During the test phase, all trajectories were paired with the same medium-intensity pain stimulus, analogous to classical conditioning studies investigating placebo and nocebo effects. As participants are still free to choose which movement to perform, the movements retain function as operant behaviors, but are no longer differentially reinforced.

We hypothesized that within each context, participants would (1) acquire lower pain expectations for trajectories paired with low vs medium vs high pain stimulation and (2) show a similar preference for performing trajectories paired with low vs medium vs high pain stimulation. Most importantly, we expected that during test, participants would (3) maintain these differential expectations and preferences when movements were no longer differentially reinforced, (4) show lower reported pain intensity for trajectories that were previously associated with low-intensity compared with medium-intensity stimuli (placebo analgesia), as well as higher reported pain intensity for trajectories that were previously associated with high-intensity compared with medium-intensity stimuli (nocebo hyperalgesia).

## 2. Methods and materials

### 2.1. Participants

Sixty participants were recruited using social media and the online departmental experiment management system. Data of 2 other participants were excluded because of equipment failure. The final sample included 58 participants (age: 21 ± 6; 38 female; 49 right handed).

Exclusion criteria were as follows: history of chronic pain, previous or current acute pain in the dominant hand, wrist, elbow, or shoulder, pregnancy, history of cardiovascular disease or respiratory disease, having a pacemaker or other implanted medical device, neurological diseases or psychiatric disorders, uncorrected hearing, or vision problems. This led to exclusion of 1 participant before the experiment. The experimental protocol was approved by the Social and Societal Ethics Committee of the KU Leuven (approval number: G- 2015 12 420). Participants received course credit or a financial compensation of €8 for their participation.

### 2.2. Apparatus and stimulus material

#### 2.2.1. HapticMaster

The HapticMaster (HM) is a 3 degrees of freedom, force-controlled robotic arm (Motekforce Link, Amsterdam, the Netherlands). The HM can be programmed to exert different forces depending on the direction in which it is moved. During the movement executed with the robotic arm, the position of the HM is recorded, enabling the use of trajectory information as a dependent variable, as well as using HM output as input for the administration of pain stimuli. The visual interface and experimental tasks were programmed in C/C++, using the Microsoft Integrated Development Environment (IDE) Visual Studio (Microsoft Corporation Redmond, WA) and the development platforms OpenGL for graphical support and HM API (Application Programming Interface), connecting to Affect 4.0 software for administration of the pain stimuli.

#### 2.2.2. Painful stimulation

The pain stimulus was delivered by a commercial stimulator (DS5; Digitimer, Welwyn Garden City, United Kingdom). Each stimulus was a 100-Hz square wave pulse lasting for 500 ms. The stimulus was delivered using 2 SensorMedics electrodes (1-cm diameter, SensorMedics, Corp., Anaheim, CA), filled with K–Y gel (Johnson & Johnson, New Brunswick, NJ), attached to the triceps tendon of the dominant arm. The maximal intensity of the painful stimulus was set individually for each participant using a single staircase calibration. During calibration, participants rated each stimulus intensity on a labeled numeric rating scale (NRS) with anchors at 0 (you feel nothing) to 10 (maximal tolerable pain). The stimulus intensity was increased to tolerance levels, until participants rated the stimulus as “painful and demanding some effort to tolerate,” corresponding to a rating of 8 on the NRS. Calibration resulted in a mean stimulus intensity of 3.71 mA (SD = 1.81, range = 1.3–10 mA), which was used as the high-intensity pain stimulus throughout the experiment. Two other pain stimulus intensities were used, which were also tailored for each participant: a low-intensity pain stimulus and a medium-intensity pain stimulus (respectively, 36% and 60% of the calibrated stimulus intensity).

### 2.3. Protocol

The experiment was conducted during a 1-hour laboratory session, consisting of a familiarization phase and 3 experimental phases. In each experimental phase, participants performed a robotic arm reaching task. On arrival to the laboratory, participants received written and oral information about the experiment and were informed that the experiment involved repeated painful electrocutaneous stimulations. Participants were free to withdraw participation at any time without any negative consequences. After providing informed consent, the electrodes for administration of the painful stimulation were attached.

#### 2.3.1. Robotic arm reaching task

**Figure 1.**
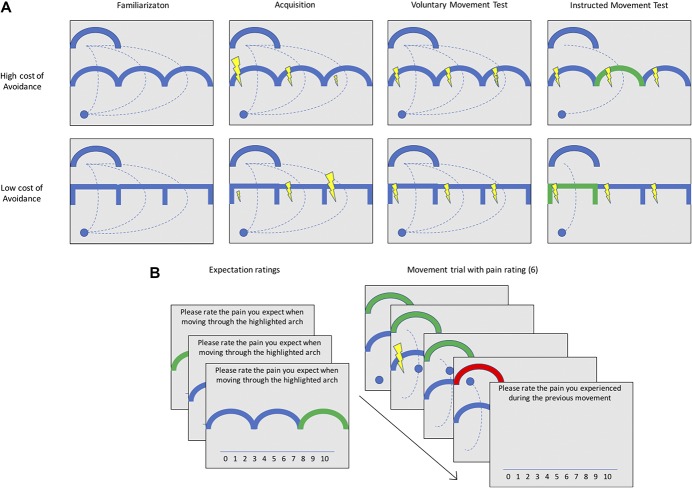
Visual representation of the experimental design using 2 different contexts across experimental phases (A), with detailed presentation of the rating scales and trial structure (B).

Participants sat in front of the HM, with the sensor of the HM positioned in front of them at shoulder height. The reaching task consisted of moving the sensor in a horizontal movement plane (0.35-m depth × 1-m radius) with the dominant hand. Real-time visual feedback of their movement was provided on a 46-inch LCD screen (36PFL3208K/12; Koninklijke Philips N.V., Amsterdam, the Netherlands). A trial counter (the number of trials remaining) was displayed at the top of the screen. Furthermore, 3 trajectory arches were positioned in the middle of the movement plane, and a target arch was placed in the upper left corner of the movement plane. From the start position, the target arch could be reached through any of these arches resulting in 3 potential movement trajectories (T1-T3) (Fig. 1).

The HM was programmed such that the tractive force exerted by the participant varied with the increasing lateral displacement of the robotic arm sensor. This resulted in lower task effort for performing T1 (shortest trajectory), compared with T2 (intermediate trajectory), and T3 (longest trajectory). All participants performed movements in 2 different experimental contexts, indicated by the use of squared vs rounded arches (Fig. 1). Visual cues further signaled the start of a trial (target arch and sensor representation changing color to green), and end of a trial (target arch color changing to red), see Figure 1, and were accompanied by a brief tone. At the end of the trial, participants released the sensor and the HM repositioned to its starting location. This was followed by an intertrial interval of 3 seconds during which the robotic arm remained fixed.

#### 2.3.2. Familiarization

The experiment started with participants being familiarized with the HM device, the experimental contexts, and the 3 possible movement trajectories, as well as practice rating pain expectation and pain intensity using a 3-pedal foot switch. During the familiarization phase, participants performed 10 movements. Assignment of context was randomized for each trial. During familiarization, no painful stimulation was administered. Participants were encouraged to explore the different possible trajectories (T1-T3) and were exposed to the differences in tractive force. At the start of the familiarization phase, the trial counter was set at 10 and counted down when the target arch was reached.

#### 2.3.3. Acquisition

The goal of the acquisition phase was to establish the different movement-pain contingencies in both contexts. Participants were free to choose which movement to perform to reach the target location. During movements, participants received electrocutaneous stimulation on each trial, when passing through one of the trajectory arches (Fig. 1). In the high cost of avoidance context, T1 (shortest and easiest movement) was followed by the high-intensity stimulus, and T3 (longest and most effortful movement) was followed by the low-intensity stimulus. In the low cost of avoidance context, T1 was followed by the low-intensity pain stimulus, and T3 was followed by the high-intensity pain stimulus. In both contexts, T2 was followed by the medium-intensity pain stimulus (Fig. 1).

Participants performed 48 trials: 4 blocks of 6 trials in both contexts. The presentation order of the blocks was semirandomized, with the restriction that both contexts occurred randomly within each sequence of 2 blocks. Each block started with a rating of pain expectation for T1-T3, and participants rated the experienced pain intensity after each trial. After pain ratings, the trial counter decreased by 1 unit.

#### 2.3.4. Voluntary movement test

The procedure during the voluntary movement test phase was similar to the acquisition phase (4 blocks of 6 trials in both contexts). However, different trajectories were no longer followed by pain stimuli of different intensities, but always followed by the medium-intensity stimulus. Participants again were able to choose which movement trajectory they wanted to perform to reach the target location. To minimize discontinuity between acquisition phase and the voluntary movement test phase, the trial counter did not reset in between phases and reached 0 at the end of the voluntary movement test phase.

#### 2.3.5. Instructed movement test

In a final exploratory phase, participants could no longer choose which movement to perform. They were prompted to perform specific movement trajectories, by highlighting 1 trajectory arch at the start of the trial (Fig. 1). This phase consisted of 24 trials, presented in 4 blocks. Within each block, each trajectory was completed twice in each context, and presentation of context and trajectory cues was randomized. At the start of each block, participants provided pain expectations for the trajectories in both of the contexts. Similar to the voluntary movement test phase, each trajectory was followed by the medium-intensity stimulus and ended with ratings of pain intensity.

#### 2.3.6. Ending the session

At the end of the instructed movement phase, participants were debriefed and were informed about the contingencies between the trajectories and pain stimuli in the different contexts and phases.

#### 2.3.7. Postexperimental questionnaires

Trait questionnaires were filled out the next day using an online survey application (LimeSurvey; LimeSurvey GmbH, Hamburg, Germany), to limit spillover effects from the experiment. Participants filled out a set of trait questionnaires, including the Positive and Negative Affect Schedule (PANAS),^10,38^ the Fear of Pain Questionnaire (FPQ),^22,30^ and the Pain Catastrophizing Scale (PCS).^31,33^ Inclusion of these questionnaires was chosen based on their associations with symptom reports and pain ratings.^8^

### 2.4. Outcome measures

Pain expectations and pain intensity were assessed using the same NRS that was used during pain calibration, anchored at 0 (you feel nothing) to 10 (maximal tolerable pain). The scale also provides anchors for 1 (you feel something, but this is not painful—merely a sensation), 2 (the sensation starts being painful, but this is a very moderate pain), and 8 (painful and demanding some effort to tolerate). For expectation ratings, participants were asked to “*Please rate the pain you expect when moving through the highlighted arch”* (Fig. 1). For pain intensity ratings, participants were asked to “*Please rate the pain you experienced during the previous movement*.” Ratings were given using a 3-pedal foot switch (USB-3FS-2; Scythe, Tokyo, Japan), which was chosen to reduce interference with the arm reaching movements. Movement trajectory choice was detected during each trial, based on the lateral position of the HM at the moment of administration of the electrocutaneous stimulus.

### 2.5. Statistical analysis overview

To present similar analyses for the pain expectation, pain intensity, and choice data, we averaged pain expectations and pain intensity data and summed movement choices for each trajectory within a block. Although all participants performed the same number of trials for each block, the operant learning procedure resulted in individual participants performing a variable number of the different trajectories in each block (range for the different trajectories being [0–6]). As repeated-measures analysis of variance is unable to deal with this type of data, we chose to average trajectory data within the same block and analyze pain expectation and intensity ratings using multilevel analysis (linear mixed-model analysis). Movement choices were analyzed using generalized (Poisson) linear mixed models. We modelled a random intercept to account for within-subject clustering and fixed effects for all other factors. Significance threshold was set at *P* < 0.05, using Satterthwaite approximation for degrees of freedom, and applied Bonferroni corrections when exploring differences in estimated marginal means. Different analyses were performed separately for each of the different experimental phases (acquisition, voluntary movement test, and instructed movement test) and outcome variables (pain expectation, pain intensity, and movement choice), and were based on models that included fixed effects of context (high vs low cost), trajectory (T1-T3), and block (1–4) (We also performed secondary analyses that included [1] main effects and interaction effects of counterbalanced context assignment [high cost-squared vs high cost-rounded], and [2] analyses that were restricted to results of right-handed individuals [n = 49], to account for potential effects of handedness on the reaching tasks. Neither of the alternative analyses changed the interpretation of the results). Confirmation of hypotheses was dependent on a significant context × trajectory interaction, which was explored further to test whether differences matched the contingencies that were presented during the acquisition phase. All analyses were performed using SPSS 24 (IBM, Corp., Armonk, NY).

## 3. Results

### 3.1. Pain expectation

#### 3.1.1. Acquisition

**Figure 2.**
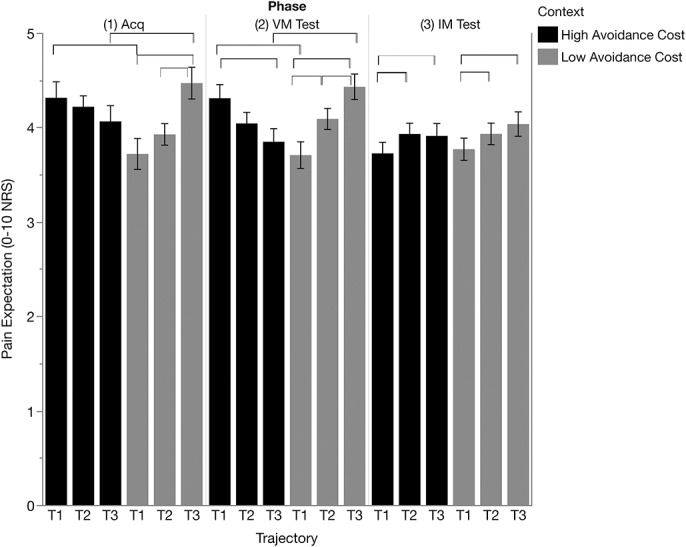
Pain expectation during the acquisition (Acq), voluntary movement test (VM Test), and instructed movement test (IM Test) phases for low cost and high cost contexts. Brackets indicate significant differences (*P* < 0.05, Bonferroni corrected). NRS, numeric rating scale.

Participants acquired pain expectations for the different trajectories in accordance with the presented movement-pain contingencies in the different contexts (T1: low cost < high cost, F(1, 1311) = 8.782, *P* = 0.003; T3 high cost < low cost, F(1, 1311) = 4.075, *P* = 0.044; context × trajectory interaction F(2, 1311) = 6.531, *P* = 0.002). Within the low cost context, participants reported higher pain expectations for T3 (paired with high-intensity stimulus) compared with T2 (paired with medium-intensity stimulus) (T3-T2 = 0.543, *P* = 0.021), but did not report lower pain expectations for T1 (paired with low-intensity stimulus) compared with T2 (T2-T1 = 0.207, *P* = 0.909). By contrast, in the high cost context, the anticipated differences in pain expectation between the different trajectories failed to reach significance (*P*-values >0.639, Fig. 2).

Block level results showed that pain expectations developed over time and differed for both contexts (block × trajectory, F(6, 1311) = 2.186, *P* = 0.042; context × block × trajectory, F(6, 1311) = 3.220, *P* = 0.004). Comparisons across contexts showed that differential pain expectations for T1 and T3 reached significance at block 2 (T1: low cost < high cost F(1,1311) = 4.075, *P* = 0.044; T3 high cost < low cost F(1,1311) = 5.580, *P* = 0.018), as well as block 4 (T1: low cost < high cost F(1,1311) = 8.281, *P* = 0.004; T3 high cost < low cost F(1,1311) = 8.035, *P* = 0.005) (see Figure S1, available at http://links.lww.com/PR9/A45). There were no other significant main effects or interactions in this analysis (all *P*-values >0.16).

#### 3.1.2. Voluntary movement test

Pain expectations showed a similar pattern to the acquisition phase, with lower pain expectations for T1 in the low cost context compared with the high cost context F(1, 1311) = 18.906, *P* < 0.001, and lower pain expectations for T3 in the high cost context compared with low cost context F(1, 1311) = 17.579, *P* < 0.001; context × trajectory F(2, 1311) = 18.295, *P* < 0.001. Furthermore, within the low cost context, participants showed significant placebo expectations (T2-T1 = 0.384, *P* = 0.017) and nocebo expectations (T3-T2 = 0.341, *P* = 0.043), whereas for the high cost context significant differences only emerged when comparing T1-T3 (0.461, *P* = 0.003, Fig. 2).

Block level results showed differences in pain expectations over time (block × trajectory F(6,1311) = 3.065, *P* = 0.006), but revealed no significant context interaction effects (all *P*-values > 0.06, see Figure S1, available at http://links.lww.com/PR9/A45).

#### 3.1.3. Instructed movement test

During the instructed movement phase, participants reported lower pain expectations for T1 compared with T2 (T1-T2 = −0.204, *P* = 0.016) or T3 (T1-T3 = −0.216, *P* = 0.011), irrespective of context (trajectory (F(2, 1311) = 5.468, *P* = 0.004), context × trajectory F(2, 1311) = 0.004, *P* = 0.996). Block level results suggested habituation across blocks (block F(3, 1311) = 7.584, *P* < 0.001). No other effects were significant (all *P*-values >0.06, see Figure S1, available at http://links.lww.com/PR9/A45).

### 3.2. Pain intensity

#### 3.2.1. Acquisition

**Figure 3.**
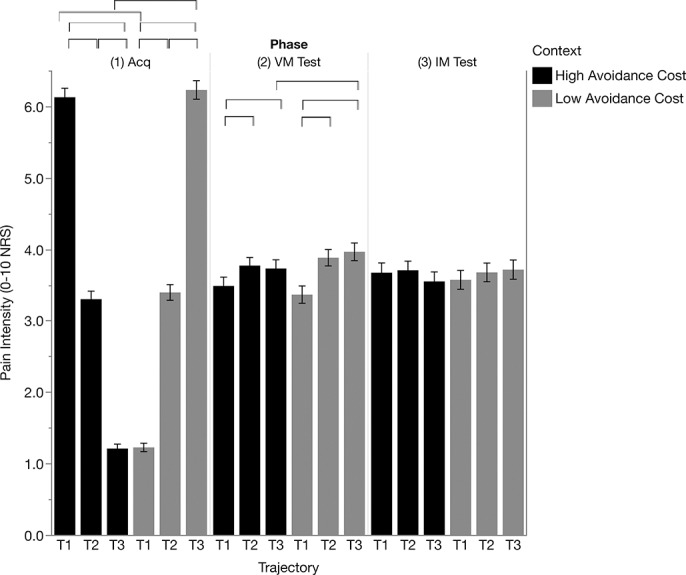
Pain intensity ratings during the acquisition (Acq), voluntary movement test (VM Test), and instructed movement test (IM Test) phases for low cost and high cost contexts. Brackets indicate significant differences (*P* < 0.05, Bonferroni corrected). NRS, numeric rating scale.

As a manipulation check, data support participants reporting different pain intensities for the low-, medium-, and high-intensity pain stimuli (Fig. 3; trajectory F(2, 1141) = 15.540, *P* < 0.001, context × trajectory F(2,1140) = 2288.505, *P* < 0.001). Block level results suggested changes in pain ratings over time (context × trajectory × block interaction F(6, 1139) = 2,150, *P* = 0.045), although pairwise comparisons across blocks did not lead to any significant differences (all *P*-values >0.065, see Figure S2, available at http://links.lww.com/PR9/A45). Other main effects or interactions did not reach significance (all *P*-values >0.14).

#### 3.2.2. Voluntary movement test

During voluntary movement test, participants reported different pain intensities in accordance with contingencies during acquisition, but only reaching significance for T3 (high cost < low cost F(1, 1198) = 7.856, *P* = 0.005), and not for T1 (high cost > low cost F(1,1198) = 1.696, *P* = 0.193) (trajectory F(2, 1002) = 26.524, *P* < 0.001) and context × trajectory (F(2, 1002) = 4.396, *P* = 0.013). Within the low cost context, participants showed a significant placebo response (T2-T1 = 0.539, *P* < 0.001) and also reported higher pain intensities for T3 than T1 (T3-T1 = 0.641, *P* < 0.001), in accordance with our expectations. However, we failed to observe a clear nocebo response (T3-T2 = 0.112, *P* = 0.093). In the high cost context, pain intensity ratings did not reflect acquired pain expectations, as pain intensity ratings were higher for T3 and T2 compared with T1 (T1-T2 = −0.345, *P* < 0.001; T1-T3 = −0.278, *P* < 0.006, T3-T2 = −0.67, *P* = 0.091) (Fig. 3). Other main effects or interactions did not reach significance (all *P*-values >0.26).

#### 3.2.3. Instructed movement test

Analysis of pain intensity ratings during the instructed movement test did not result in any significant main effects or interaction effects (all *P*-values >0.33).

### 3.3. Movement choices

#### 3.3.1. Acquisition

**Figure 4.**
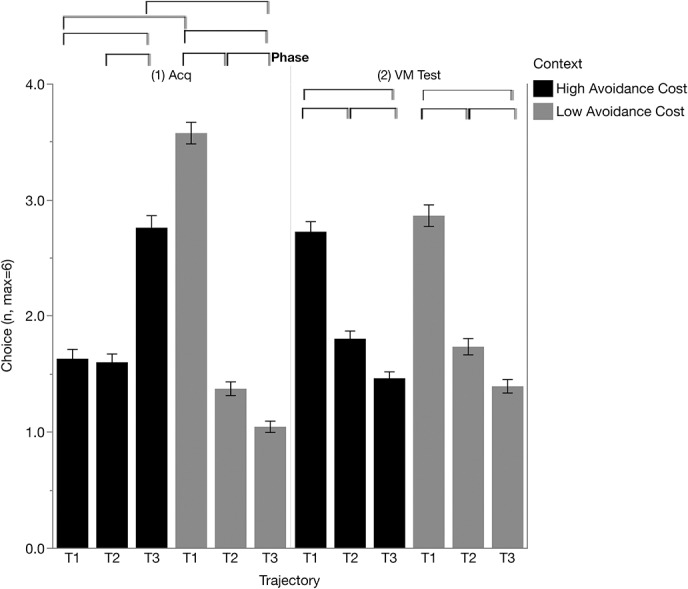
Movement trajectory choice during the acquisition (Acq) and voluntary movement test (VM Test) phases for low cost and high cost contexts. Brackets indicate significant differences (*P* < 0.05, Bonferroni corrected).

Participants showed movement choices in accordance with the presented stimulus intensities preference for T1: low cost > high cost, F(1, 1368) = 169.19, *P* < 0.001; preference for T3: high cost > low cost, F(1, 1368) = 177.65, *P* < 0.001; and trajectory (F(2, 1368) = 54.668, *P* < 0.001), context (F(1, 1368) = 7.134, *P* < 0.001), and context × trajectory (F(2, 1368) = 164.309, *P* < 0.001). Within the low cost context, there was a preference for T1 movements compared with T2 (T1-T2 = 2.201, *P* < 0.001) and T3 (T1-T3 = 2.528, *P* < 0.001), as well as avoidance of T3 movements compared with T2 (T3-T2 = −0.327, *P* = 0.001). In the high cost context, participants showed a clear preference for T3 movements (T3-T2 = 1.156, *P* < 0.001; T3-T1 = 1.131, *P* < 0.001), but there was no significant difference in choice between T1 and T2 movements (T2-T1 = −0.025, *P* = 0.830) (Fig. 4).

Block level results suggested that these preferences were present early on during acquisition and did not change across blocks, see Figure S3 (available at http://links.lww.com/PR9/A45), context × block × trajectory interaction (F(6, 1368) = 1.243, *P* = 0.281). There were no other significant main effects or interaction effects (all *P*-values >0.49).

#### 3.3.2. Voluntary movement test

During the voluntary movement test, movement preferences no longer differed by context (T1: low cost > high cost, F(1, 1368) = 0.763, *P* = 0.383; T3: high cost > low cost, F(1, 1368) = 0.395, *P* = 0.530; and context × trajectory F(2, 1368) = 0.736, *P* = 0.479). For both contexts, participants had a clear preference for T1 compared with T2 and to T2 compared with T3 (all *P*-values <0.006, Fig. 4; main effect of trajectory F(2, 1368) = 113.013, *P* < 0.001). Block level results showed that differences between T1 and T2 as well as T1 and T3 were present from the start of the voluntary movement test, whereas differences between T2 and T3 did not emerge until block 3, see Figure S3 (available at http://links.lww.com/PR9/A45), block × trajectory (F(6, 1368) = 2.369, *P* = 0.028). There were no other significant main effects or interactions (all *P*-values >0.47).

## 4. Discussion

In this study, we investigated the acquisition of movement choices, pain expectations, and pain modulation in response to movement-pain contingencies. Participants performed an operant learning task in 2 contexts. In 1 context, avoidance responses were associated with high cost, while in the other, context avoidance was less costly. Similar to other placebo/nocebo classical conditioning procedures, participants were subjected to contingencies between specific actions and changes in pain intensity. In addition, similar to other operant learning procedures, participants were able to change actions in response to the experienced contingencies. Irrespective of context, the results revealed that pain expectations and movement choices can be readily acquired through operant learning procedures. These findings are in line with current models of pain-related disability, which posit that individuals learn to avoid situations that are expected to cause pain or harm,^35^ as well as recent findings showing that operant learning tasks can be used to model pain-related fear and avoidance behavior.^23^ Similar to other placebo/nocebo experiments, acquired pain expectations do not fully match experienced pain intensities.^4^ Furthermore, participants do not fully avoid painful movements, suggesting continued exploration of movement-pain contingencies.^18^

During the test phase, participants reported different pain intensities across contexts in the absence of differential reinforcement, although this was limited to the most effortful movement (T3). Furthermore, within-context patterns of pain modulation only matched acquired expectations in the low cost context, in which we only observed a placebo effect, but no nocebo effect. Nevertheless, the resulting pain modulation was similar in magnitude to placebo effects, resulting from classical conditioning procedures without co-occurring instruction,^21^ and shows that placebo pain modulation extends to information that has been acquired through an operant learning procedure. Other studies have shown pain modulation using different operant procedures, including positive or negative verbal reinforcement,^14,19,20^ as well as monetary rewards and punishments,^11^ but only a limited number of studies have shown that self-reported pain can be changed using pain as a reinforcer. Exceptions include studies by Becker et al.,^1,2^ who have shown that operant conditioning using temporary pain increases/decreases can be used to elicit habituation and sensitization of pain. Contrary to these studies, we did not reinforce or punish verbal pain behavior, but pain-related movements, and investigated the impact of these movements on pain intensity ratings. This is procedurally similar to a recent study by Jung et al.,^15^ in which operant learning (choice between 2 treatments of different efficacy) elicited placebo effects in a treatment context (compared with instructed no treatment trials), but no differences in pain reduction were observed between the placebo treatments.

Our study complements these findings by showing pain modulation in the context of instrumental avoidance behavior, thereby indicating that movements can acquire pain-modulating properties due to previous operant learning. We found that after being followed by pain reduction in an operant learning task, performing these movements elicited pain reduction. The voluntary nature of these movements differentiates our task from classical conditioning procedures that have previously been applied in placebo research. Although the mechanisms of operant learning procedures deserve further investigation,^9^ these findings provide a procedural analog to real-life operant learning in the context of pain. The focus on voluntary movements is especially relevant for individuals suffering from pain, who may avoid movements that elicit pain or alter behavior to seek safety when experiencing pain.^32^ Furthermore, recent findings show a feedback loop between expectations and subsequent (placebo or nocebo) pain effects,^13^ suggesting a potential pathway for the development and maintenance of pain-related safety behaviors.

The large number of operant test trials enabled us to investigate the persistence of pain expectations, pain and movement choice modulations despite changed contingencies. Interestingly, pain expectations and pain intensity reports showed stronger persistence compared with movement choice, which quickly responded to a lack of reinforcement after acquisition. The quick change in movement choices is unexpected, as avoidance behaviors are quite resistant to extinction.^17,26^ Furthermore, previous research showed much slower changes in avoidance behaviors during extinction of pain-related fear using a similar experimental setup.^23^ Differences in the research design—the use of a between-participant (yoked) control group instead of within-participant controls—may partly explain these observed differences (see further discussion of limitation).

The fear-avoidance model of chronic pain proposes that persistence of expectations and pain modulation are maintained by continued avoidance behavior.^16,37^ Our findings do not fit this proposed pathway and suggest that pain expectations and pain modulation can persist in the absence of avoidance behavior. This persistence is consistent with a lack of extinction of pain modulation and expectations observed in other placebo and nocebo studies^4,7^ and could contribute to the experienced burden of acute or chronic pain. Furthermore, our findings suggest that exposure treatment for chronic pain—aimed at reducing fear of pain and pain-related avoidance behavior—may be hampered by persisting pain modulation during exposure to these feared behaviors.

During the instructed movement test phase, we no longer observed differential pain expectations or pain modulation across context, suggesting extinction of these responses. The differences between both test phases (voluntary vs instructed movements; blocked vs random presentation of context cues) hinders the interpretation of this finding.

Unexpectedly, and of interest, is that the cost of avoidance behavior moderated the magnitude of differences in expectations and pain modulation. During the voluntary movement test, we observed evidence for both placebo and nocebo expectations in the low cost context, whereas these effects were weaker in the high cost context. The pattern of expectations was matched with similar effects on pain modulation in the low cost context, but not for the high cost context. Context differences in choice behavior during acquisition suggest that movement trajectory choices not only depended on movement-pain contingencies but also reflected their relative cost. The resulting differences in experience with movement-pain contingencies may have led to a difference in the precision of pain expectations, which in turn may have led to differences in pain modulation.^3^

However, these findings may also be the result of a confound induced by differences in the physical properties of the arm reaching movements. Alternatively, more difficult movements may have had different impact on endogenous pain modulation or may have distracted participants from pain.^27,28^ These differences may have led participants to observe contingencies between the reaching movements and pain intensities differently. For example, participants may have extracted a rule, such as “more difficult movements lead to more pain.”^39^ Taken together, this suggests that in the low cost context, experiences during acquisition and voluntary movement test may have been more similar and consistent across different features, which has been suggested to increase the probability of a placebo effect.^12^

Finally, differences in movement choices during the voluntary movement test could have contributed to the observed differences in expectations and pain modulation. In this phase, movement choices were similar for both contexts, with a stronger preference for less effortful (T1) compared with more effortful (T3) movements. These differences could result in different levels of exposure to the novel pain-movement contingencies, which would explain why for the most chosen movement (T1) pain intensity ratings did not differ across contexts, whereas pain intensity ratings still differed for the least chosen movement (T3).

By varying movement-pain contingencies across contexts in a within-subject design, we were able to control for physical properties of the arm reaching movements across context. However, our design holds a risk of carryover effects or even may have confused participants, who may have based their ratings on experimental cues (eg, the specific movement trajectory or associated force), without incorporating information about the experimental context. Moreover, the exposure to different outcomes for the same movement trajectory during acquisition may already have introduced participants to the idea that outcomes for a specific movement trajectory can differ or change throughout the experiment, which may explain some of our null results during the voluntary movement test phase.

A further limitation of this study is that the magnitude of pain modulation was relatively small. Although the observed magnitude is within range of studies using classical conditioning without explicit placebo/nocebo instruction,^21^ it is uncertain whether these effects could contribute to meaningful differences in the daily life of individuals suffering from pain. A final limitation is that pain expectation was only assessed at the start of each block, whereas pain intensity and movement choices were measured on each trial. This limits drawing conclusions on changes in expectation that may have occurred within a block as well as make strong inferences about the interdependence of these different pain-related outcomes.

Despite these limitations, this study shows that while performing an operant learning task, participants learn to expect and avoid pain. During test phases, changes in reinforcement quickly changed pain-related behaviors. Changes in pain expectations were maintained after disconfirmation and co-occurred with pain modulation, suggesting difficulties in changing pain-related expectations and their lasting impact on pain.

## Disclosures

The authors have no conflict of interest to declare.

This research was supported by the “Asthenes” long-term structural funding Methusalem grant by the Flemish Government, Belgium. A. Meulders is a postdoctoral researcher of the Research Foundation Flanders (FWO-Vlaanderen), Belgium (grant ID 12E3717N), and is supported by a Vidi grant from the Netherlands Organization for Scientific Research (NWO), the Netherlands (grant ID 452-17-002).

Portions of the article have been presented at the SIPS conference on placebo studies, Leiden, the Netherlands, April 2–4, 2017, and the International Congress of Behavioral Medicine, Santiago de Chile, Chile, November 14–17, 2018.

## Supplementary Material

SUPPLEMENTARY MATERIAL

## Appendix A. Supplemental digital content

Supplemental digital content associated with this article can be found online at http://links.lww.com/PR9/A45.

## References

[1] Becker S Kleinböhl D Baus D Hölzl R Operant learning of perceptual sensitization and habituation is impaired in fibromyalgia patients with and without irritable bowel syndrome. PAIN 2011;152:1408–17. 21439728 10.1016/j.pain.2011.02.027

[2] Becker S Kleinböhl D Klossika I Hölzl R Operant conditioning of enhanced pain sensitivity by heat–pain titration. PAIN 2008;140:104–14. 18774227 10.1016/j.pain.2008.07.018

[3] Büchel C Geuter S Sprenger C Eippert F Placebo analgesia: a predictive coding perspective. Neuron 2014;81:1223–39. 24656247 10.1016/j.neuron.2014.02.042

[4] Colagiuri B Quinn VF Colloca L Nocebo hyperalgesia, partial reinforcement, and extinction. J Pain 2015;16:995–1004. 26168876 10.1016/j.jpain.2015.06.012

[5] Colagiuri B Schenk LA Kessler MD Dorsey SG Colloca L The placebo effect: from concepts to genes. Neuroscience 2015;307:171–90. 26272535 10.1016/j.neuroscience.2015.08.017 PMC5367890

[6] Colloca L Grillon C Understanding placebo and nocebo responses for pain management. Curr Pain Headache Rep 2014;18:419. 24771206 10.1007/s11916-014-0419-2 PMC4142751

[7] Colloca L Sigaudo M Benedetti F The role of learning in nocebo and placebo effects. PAIN 2008;136:211–18. 18372113 10.1016/j.pain.2008.02.006

[8] Corsi N Colloca L Placebo and nocebo effects: the advantage of measuring expectations and psychological factors. Front Psychol 2017;8:308. 28321201 10.3389/fpsyg.2017.00308 PMC5337503

[9] De Houwer J A functional-cognitive perspective on the relation between conditioning and placebo research. In: Colloca L, editor. Neurobiology of the placebo effect, Part I. Vol. 138: Academic Press, 2018 p. 95–111. Available at: http://hdl.handle.net/1854/LU-8547649. Accessed February 7, 2019. 10.1016/bs.irn.2018.01.007 29681337

[10] Engelen U De Peuter S Victoir A Van Diest I Van den Bergh O Verdere validering van de “Positive and Negative Affect Schedule” (PANAS) en vergelijking van twee Nederlandstalige versies (Further validation of the Positive and Negative Affect Schedule (PANAS) and comparison of two Dutch versions). Gedrag Gezondheid 2006;34:89–102.

[11] Flor H Knost B Birbaumer N The role of operant conditioning in chronic pain: an experimental investigation. PAIN 2002;95:111–18. 11790473 10.1016/s0304-3959(01)00385-2

[12] Geuter S Koban L Wager TD The cognitive neuroscience of placebo effects: concepts, predictions, and physiology. Annu Rev Neurosci 2017;40:167–88. 28399689 10.1146/annurev-neuro-072116-031132

[13] Jepma M Koban L Van Doorn J Jones M Wager TD Behavioural and neural evidence for self-reinforcing expectancy effects on pain. Nat Hum Behav 2018;2:838. 10.1038/s41562-018-0455-8 PMC6768437 31558818

[14] Jolliffe CD Nicholas MK Verbally reinforcing pain reports: an experimental test of the operant model of chronic pain. PAIN 2004;107:167–75. 14715403 10.1016/j.pain.2003.10.015

[15] Jung WM Lee YS Wallraven C Chae Y Bayesian prediction of placebo analgesia in an instrumental learning model. PLoS One 2017;12:e0172609. 28225816 10.1371/journal.pone.0172609 PMC5321416

[16] Kroska EB A meta-analysis of fear-avoidance and pain intensity: the paradox of chronic pain. Scand J Pain 2016;13:43–58. 28850534 10.1016/j.sjpain.2016.06.011

[17] Krypotos AM Effting M Kindt M Beckers T Avoidance learning: a review of theoretical models and recent developments. Front Behav Neurosci 2015;9:189. 26257618 10.3389/fnbeh.2015.00189 PMC4508580

[18] Lenow JK Constantino SM Daw ND Phelps EA Chronic and acute stress promote overexploitation in serial decision making. J Neurosci 2017;37:5681–9. 28483979 10.1523/JNEUROSCI.3618-16.2017 PMC5469305

[19] Linton SJ Götestam KG Controlling pain reports through operant conditioning: a laboratory demonstration. Percept Mot Skills 1985;60:427–37. 4000858 10.2466/pms.1985.60.2.427

[20] Lousberg R Groenman NH Schmidt AJ Gielen AA Operant conditioning of the pain experience. Percept Mot Skills 1996;83:883–900. 8961326 10.2466/pms.1996.83.3.883

[21] Madden VJ Harvie DS Parker R Jensen KB Vlaeyen JWS Moseley GL Stanton TR Can pain or hyperalgesia be a classically conditioned response in humans? A systematic review and meta-analysis. Pain Med 2015:pnv044. 10.1093/pm/pnv044 26814278

[22] McNeil DW Rainwater AJ Development of the fear of pain questionnaire-III. J Behav Med 1998;21:389–410. 9789168 10.1023/a:1018782831217

[23] Meulders A Franssen M Fonteyne R Vlaeyen JWS Acquisition and extinction of operant pain-related avoidance behavior using a 3 degrees-of-freedom robotic arm. PAIN 2016;157:1094–104. 26761388 10.1097/j.pain.0000000000000483

[24] Meulders A Vansteenwegen D Vlaeyen JWS The acquisition of fear of movement-related pain and associative learning: a novel pain-relevant human fear conditioning paradigm. PAIN 2011;152:2460–9. 21723664 10.1016/j.pain.2011.05.015

[25] Meulders A Vlaeyen JWS The acquisition and generalization of cued and contextual pain-related fear: an experimental study using a voluntary movement paradigm. PAIN 2013;154:272–82. 23211100 10.1016/j.pain.2012.10.025

[26] Mowrer OH A stimulus-response analysis of anxiety and its role as a reinforcing agent. Psychol Rev 1939;46:553–65.

[27] Nijs J Kosek E Van Oosterwijck J Meeus M Dysfunctional endogenous analgesia during exercise in patients with chronic pain : to exercise or not to exercise? Pain Physician 2012;15:ES205–ES213. 22786458

[28] Pera DL Brancucci A Armas LD Percio CD Miliucci R Babiloni C Restuccia D Rossini PM Valeriani M Inhibitory effect of voluntary movement preparation on cutaneous heat pain and laser-evoked potentials. Eur J Neurosci 2007;25:1900–7. 17432974 10.1111/j.1460-9568.2007.05389.x

[29] Petersen GL Finnerup NB Colloca L Amanzio M Price DD Jensen TS Vase L The magnitude of nocebo effects in pain: a meta-analysis. PAIN 2014;155:1426–34. 24780622 10.1016/j.pain.2014.04.016 PMC4213146

[30] Roelofs J Peters ML Deutz J Spijker C Vlaeyen JWS The Fear of Pain Questionnaire (FPQ): further psychometric examination in a non-clinical sample. PAIN 2005;116:339–46. 15979794 10.1016/j.pain.2005.05.003

[31] Sullivan MJ Bishop SR Pivik J The pain catastrophizing scale: development and validation. Psychol Assess 1995;7:524.

[32] Tang NKY Salkovskis PM Poplavskaya E Wright KJ Hanna M Hester J Increased use of safety-seeking behaviors in chronic back pain patients with high health anxiety. Behav Res Ther 2007;45:2821–35. 17588530 10.1016/j.brat.2007.05.004

[33] Van Damme S Crombez G Bijttebier P Goubert L Van Houdenhove B A confirmatory factor analysis of the Pain Catastrophizing Scale: invariant factor structure across clinical and non-clinical populations. PAIN 2002;96:319–24. 11973004 10.1016/S0304-3959(01)00463-8

[34] Vase L Petersen GL Riley JL Price DD Factors contributing to large analgesic effects in placebo mechanism studies conducted between 2002 and 2007. PAIN 2009;145:36–44. 19559529 10.1016/j.pain.2009.04.008

[35] Vlaeyen JWS Learning to predict and control harmful events: chronic pain and conditioning. PAIN 2015;156:S86–93. 25789440 10.1097/j.pain.0000000000000107

[36] Vlaeyen JWS Crombez G Linton SJ The fear-avoidance model of pain. PAIN 2016;157:1588–9. 27428892 10.1097/j.pain.0000000000000574

[37] van Vliet CM Meulders A Vancleef LMG Vlaeyen JWS The opportunity to avoid pain may paradoxically increase fear. J Pain 2018;19:1222–30. 29777952 10.1016/j.jpain.2018.05.003

[38] Watson D Clark LA Tellegen A Development and validation of brief measures of positive and negative affect: the PANAS scales. J Pers Soc Psychol 1988;54:1063–70. 3397865 10.1037//0022-3514.54.6.1063

[39] Wong AHK Lovibond PF Excessive generalisation of conditioned fear in trait anxious individuals under ambiguity. Behav Res Ther 2018;107:53–63. 29870830 10.1016/j.brat.2018.05.012

