# Supplementary material for: Placebo and nocebo effects and operant pain-related avoidance learning
Source: Pain Rep. 2019 Jun 7;4(3):e748. doi: 10.1097/PR9.0000000000000748 (PMC6749895; doi:10.1097/PR9.0000000000000748)
Supplement: SUPPLEMENTARY MATERIAL [file painreports-4-e748-s001.docx]

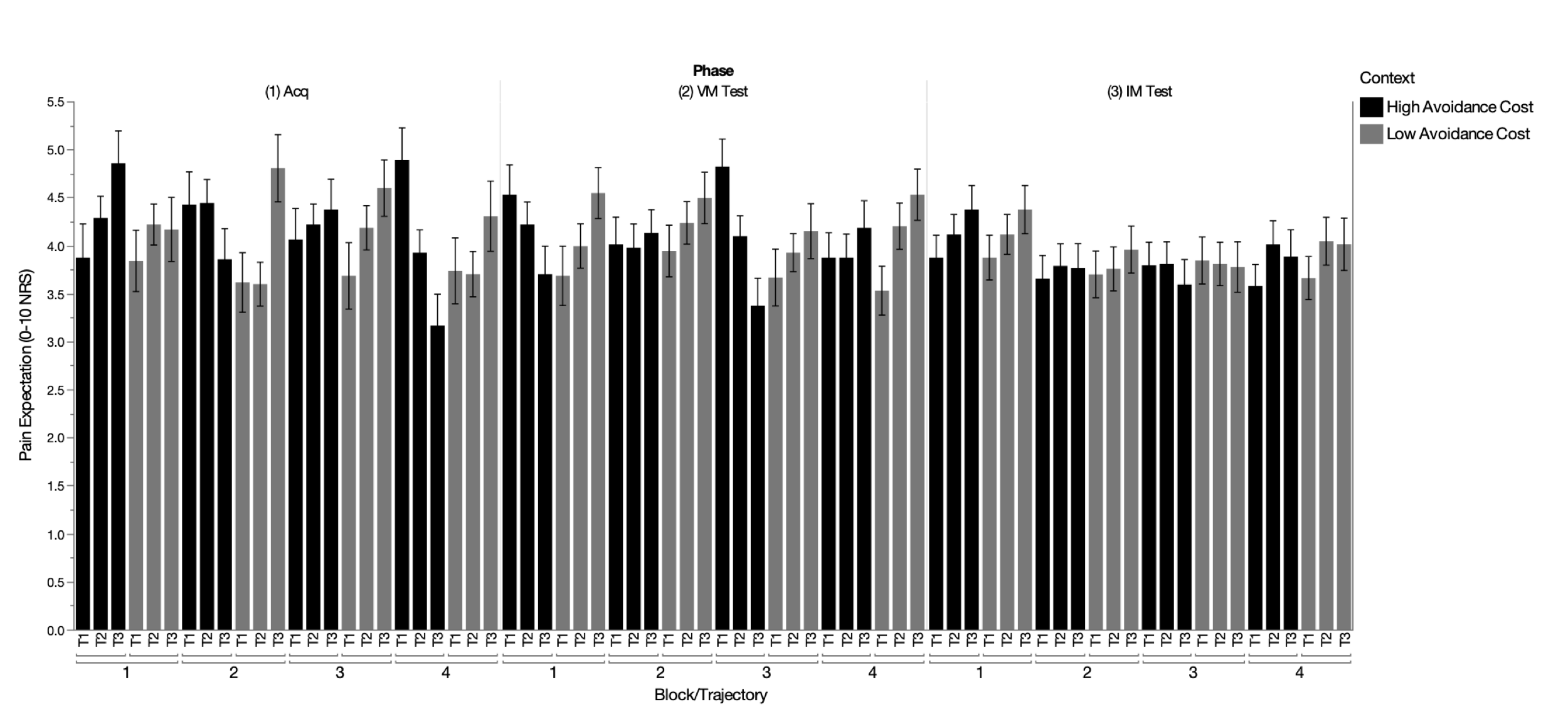


Supplementary Figure S1: Pain Expectation across Blocks during the Acquisition (Acq), Voluntary Movement Test (VM Test), and Instructed Movement Test (IM Test) phases for Low cost and High cost contexts.


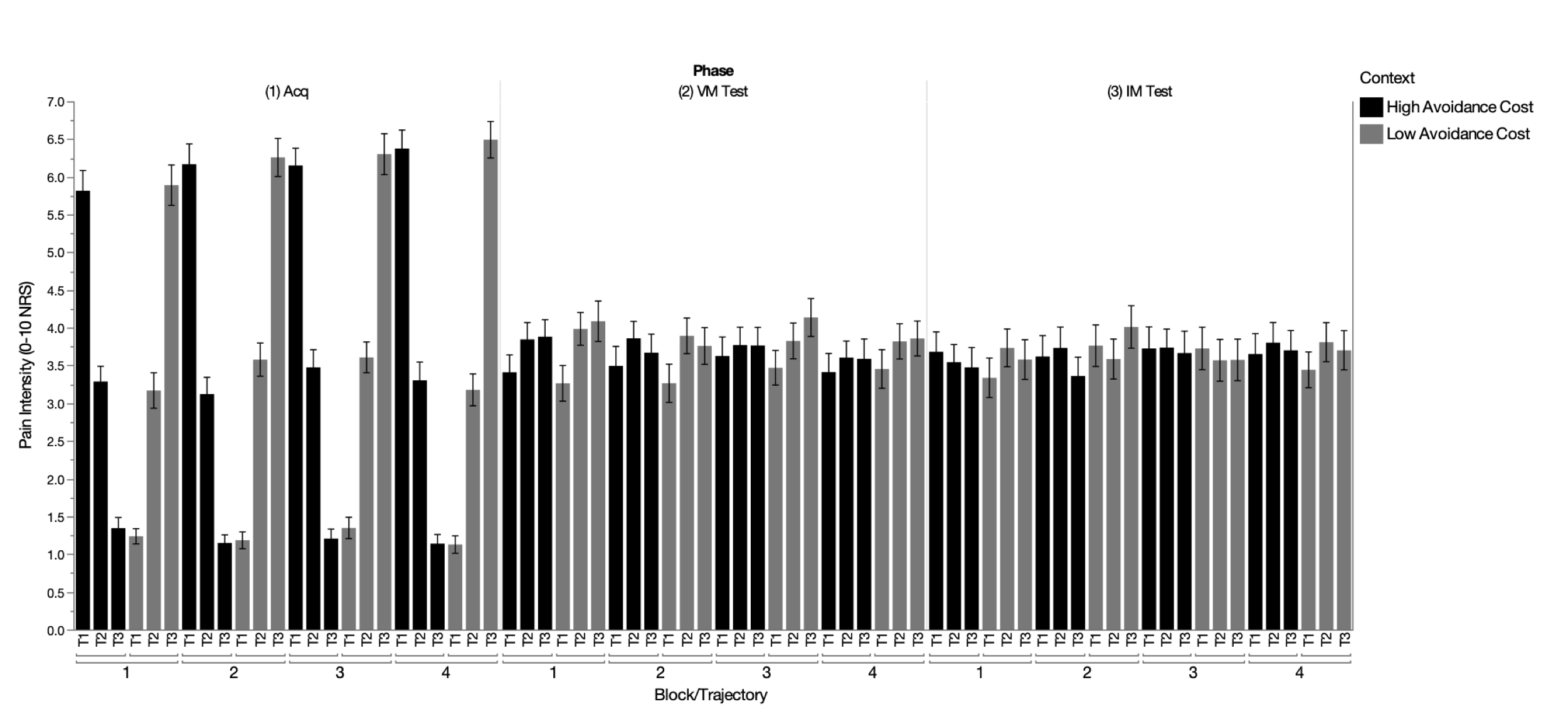


Supplementary Figure S2: Pain Intensity ratings across Blocks during the Acquisition (Acq), Voluntary Movement Test (VM Test), and Instructed Movement Test (IM Test) phases for Low cost and High cost contexts.


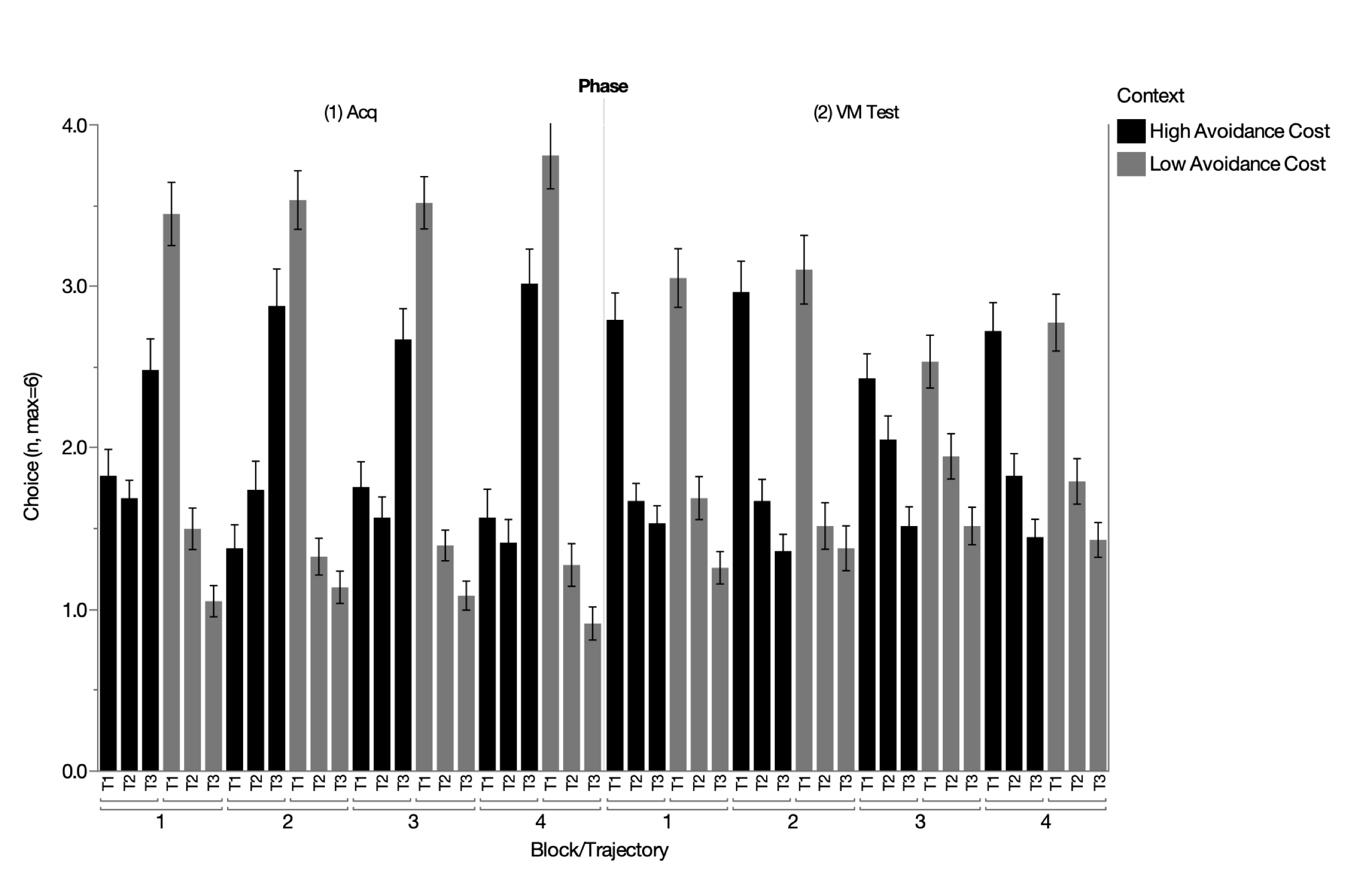


Supplemental Figure S3: Movement trajectory choice across Blocks during the Acquisition (Acq) and Voluntary Movement Test (VM Test) phases for Low cost and High cost contexts.
